# Uncommon disseminated muscular metastasis from suspected lung adenocarcinoma in a 18F-FDG PET/CT study

**DOI:** 10.1186/s41824-022-00143-4

**Published:** 2022-10-24

**Authors:** S. Pacella

**Affiliations:** Nuclear Medicine Department, ASST Ovest Milanese, Ospedale Civile Di Legnano, Milan, Italy

**Keywords:** Lung cancer, PET/CT, Muscle metastasis, Detection

## Abstract

Skeletal muscle metastasis from lung cancer is a rare phenomenon. In this case report, FDG PET/CT imaging detected unexpected metastatic spread in skeletal muscles from lung cancer.

In lung cancer, the multiple locations frequent metastases are represented by the adrenal glands, the liver, the skeleton and the brain. In this sense, PET certainly has an advantage compared to other imaging methods because the oncological tomoscintigraphic study is always performed with a total body scan (from the skull base to the proximal third of the femur) and therefore also allows an evaluation of all body districts, with the exception of the brain, to search for remote locations of the disease (M staging). Several studies have demonstrated the ability of PET to identify distant metastases with high specificity, superior to what can be achieved with conventional imaging, including CT (Baum et al. 2004).

Some authors have shown that in patients with conventional negative imaging, PET found occult metastases in 5–29% of cases. The diagnostic impact of the method has a cascade impact on patient management: Many studies have demonstrated the ability of PET to change the stage of the disease in 27–62% of cases and consequently the management of the patient in varying percentages between 25% and about 50% especially in the choice of the type of treatment (curative vs palliative) (Schrevens and Lorent 2004).

In this case report, FDG PET/CT imaging detected unexpected metastatic spread in skeletal muscles from lung cancer.

A 51-year-old man with multiple subcutaneous nodular lesions of suspected oncological origin referred to our Nuclear Medicine Department in order to find out the culprit primary lesion.

The 18F-FDG PET/CT study demonstrated an increased FDG uptake in the primary tumor located in the upper lobe of the left lung, bilateral adrenal glands metastasis, multiple osteolytic bone lesions and multiple hypermetabolic nodular lesions in various skeletal muscle (Figs. 1, 2, 3), also of the tongue muscles (Fig. 4).

**Fig. 1:**
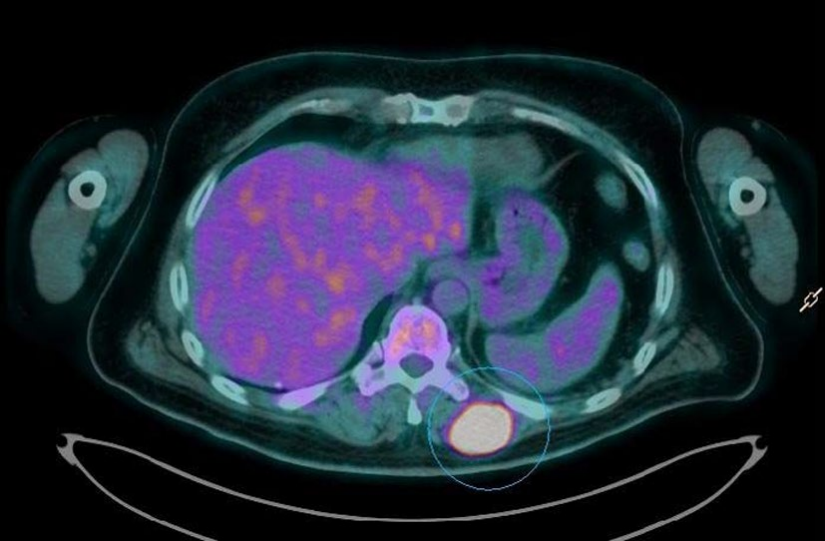
Multiple hypermetabolic nodular lesions in various skeletal muscle (image 1)

**Fig. 2:**
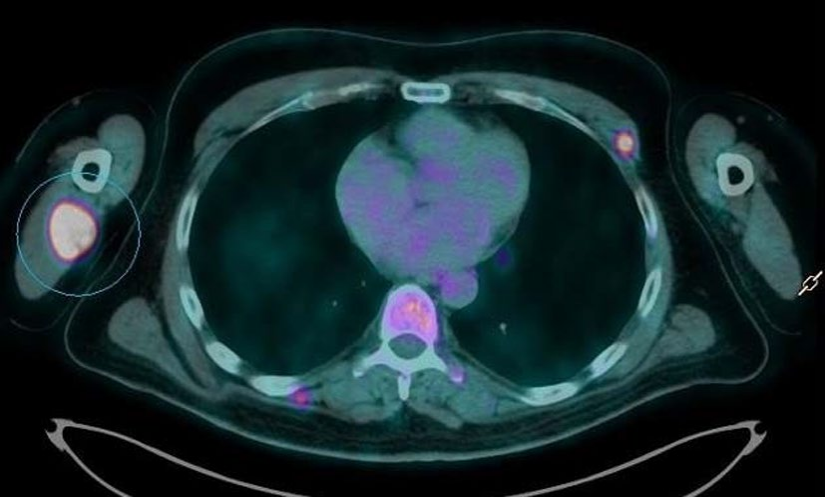
Multiple hypermetabolic nodular lesions in various skeletal muscle (image 2)

**Fig. 3:**
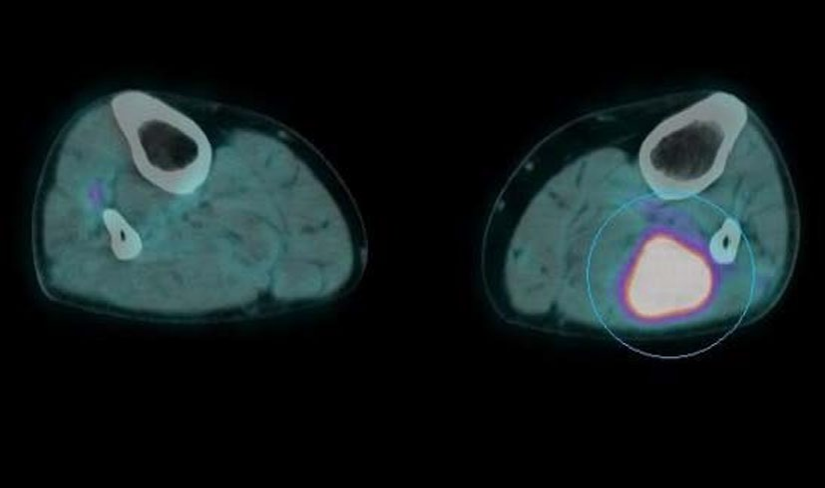
Multiple hypermetabolic nodular lesions in various skeletal muscle (image 3)

**Fig. 4:**
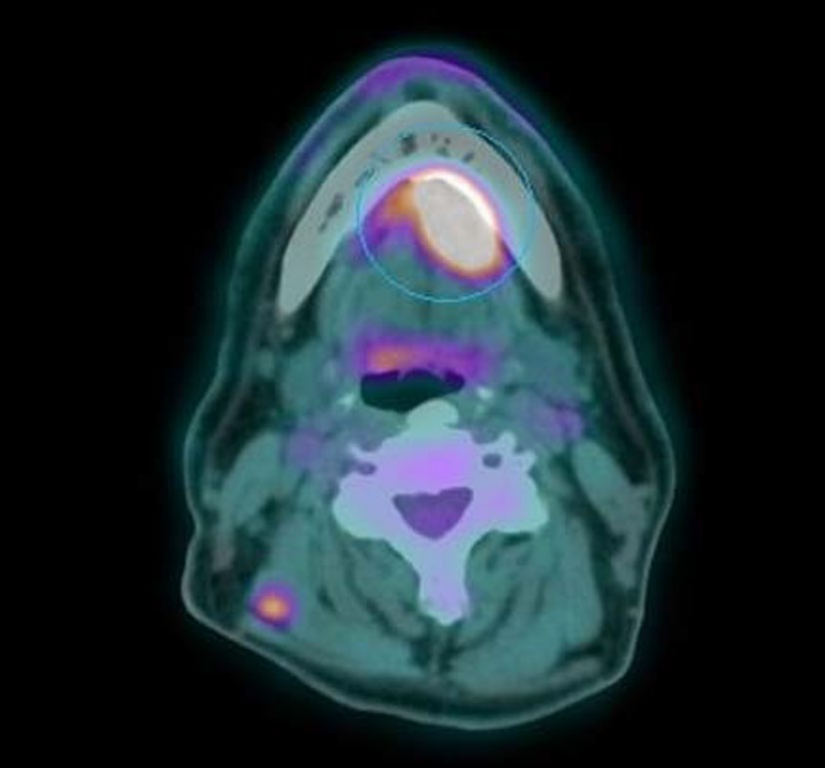
Metastatic hypermetabolic lesion of the tongue muscles

Surgical biopsy from hypermetabolic muscle mass in the posteroinferior thoracic wall revealed to be a metastatic site from adenocarcinoma of lung origin staining with cytokeratin-7, but thyroid transcription factor-1 and cytokeratin-20 were negative. Also thyroid transcription factor-1 was negative, and this factor may occur in 28% of cases with lung adenocarcinoma (Yatabe et al. 2002) (Fig. 5).

**Fig. 5:**
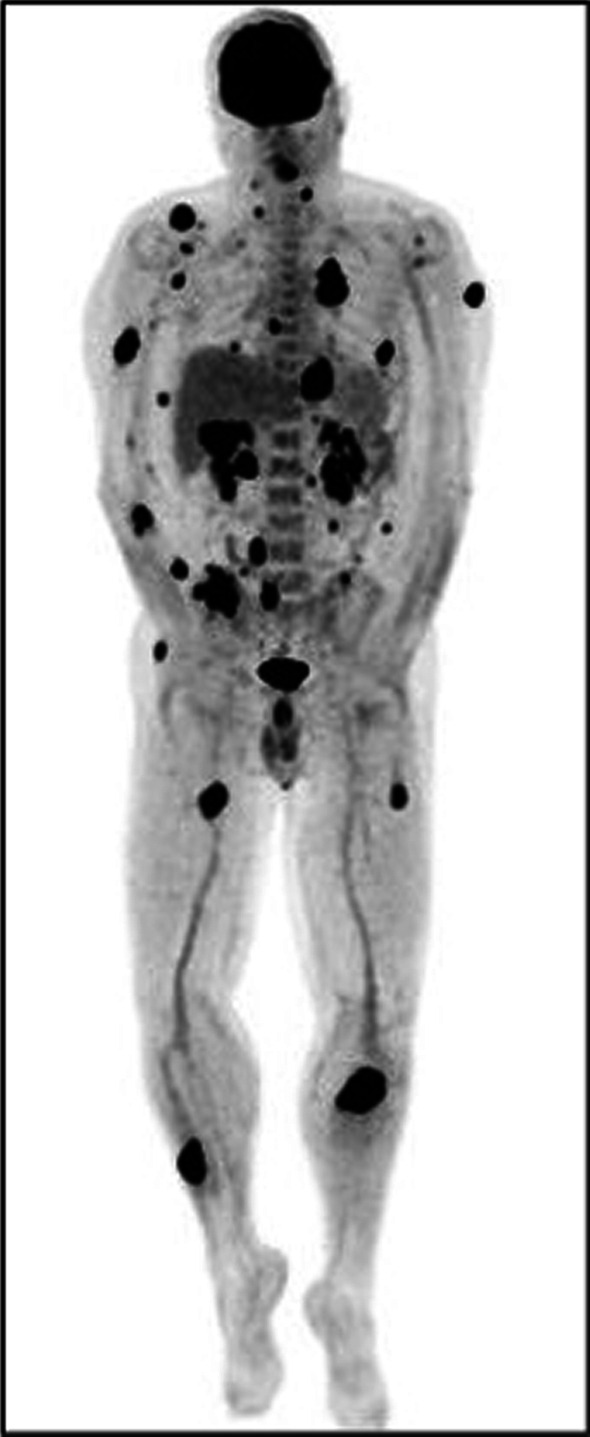
MIP image of 18F-FDG PET/CT study

## Conclusions

FDG PET/CT scan in this case demonstrated to be an excellent imaging method in detecting muscle involvements in lung cancer patients, which are a rare site of metastatic involvement from adenocarcinoma lung cancer (Giorgio et al. 2004; Kaira et al. 2009).

## Data Availability

Not applicable.

## References

[CR1] Baum RP, Hellwig D, Mezzetti M (2004). Position of nuclear medicine modalities in the diagnostic workup of cancer patients: lung cancer. Q J Nucl Med Mol Imaging.

[CR2] Di Giorgio A, Sammartino P, Cardini CL (2004). Lung cancer and skeletal metastases. Ann Thorac Surg.

[CR3] Kaira K, Ishizuka T, Yanagitani N (2009). Forearm muscle metastasis as an initial clinical manifestation of lung cancer. South Med J.

[CR4] Schrevens L, Lorent N, Dooms C (2004). The role of PET scan in diagnosis, staging and management of non-small cell lung cancer. Oncologist.

[CR5] Yatabe Y, Mitsudomi T, Takahashi T (2002). TTF-1 expression in pulmonary adenocarcinomas. Am J Surg Pathol.

